# Qualia as control-theoretic constructs for autonomous agents: event phase space as an action-oriented semantic safety layer

**DOI:** 10.3389/frobt.2026.1811996

**Published:** 2026-05-29

**Authors:** Vladimir Suvorov

**Affiliations:** Research Computing Center, Lomonosov Moscow State University, Moscow, Russia

**Keywords:** attractor dynamics, autonomous agents, CARLA simulation, control barrier functions, embodied AI, event phase space, neural oDE, qualia

## Abstract

Autonomous systems face a fundamental complexity barrier in open-world environments: decomposing scenes into discrete entities and searching over combinatorial action spaces leads to prohibitive computational costs, yet biological agents navigate equivalent environments in real time through holistic situational assessments — states that the phenomenological tradition terms qualia. This paper argues the gap is architectural and introduces three contributions. First, we propose that qualia are operationally indicated by assessment structures characterized by geometric coherence on a manifold, multimodality, joint encoding of state and rate, action-readiness, and intrinsic predictive content, when instantiated in an AI entity satisfying explicit subsistence and non-identity conditions. Second, we introduce the event phase space: a differentiable manifold of substantially reduced dimensionality compared to World Models, endowed with learned dynamics in which safe modes correspond to stable attractors and safety boundaries to separatrices, thereby replacing combinatorial search with gradient navigation at O(n) complexity. Third, the theoretical model is transferred to a standard industry benchmark through validation in CARLA Parked Vehicle Occlusion scenarios (1,000 episodes per controller, 5 seeds), where an alarm function derived purely from the learned vector field achieves AUC = 0.812 and reduces the collision rate by 29.6% in stress scenarios (p = 0.0034), outperforming TTC-based pipelines by 60× while operating at 1.9 ms/decision on CPU with only 6,550 parameters — substantially fewer than comparable latent-CBF methods.

## 1 Introduction

Autonomous robotics and self-driving systems have achieved substantial progress in structured environments, yet remain fundamentally limited in open-world scenarios. The core limitation is architectural: many contemporary systems still decompose scenes into discrete entities, predict their trajectories separately, and search over large action spaces, causing computational cost to grow rapidly with scene richness (Yurtsever et al., 2020).

Control architectures have evolved toward increasingly complete accommodation of environmental complexity and progressively stricter requirements on goal attainment, safety, and human-centricity. Early finite-state controllers operated reliably only within fully specified state spaces. Reactive architectures introduced direct sensorimotor coupling, but at the cost of weak anticipatory capacity (Brooks, 1991). Model-based planning added explicit world representations, yet planning complexity remained a bottleneck in dynamic multi-agent environments (Schwarting et al., 2018).

A major shift occurred with the introduction of learned latent world models, which compress high-dimensional observations into predictive internal states that can support planning and control (Ha and Schmidhuber, 2018). Subsequent work has significantly extended this paradigm toward more general and scalable settings (Hafner et al., 2023). In parallel, continuous latent dynamics have been formalized through neural ordinary differential equations, enabling compact representations of system evolution (Chen et al., 2018). Safety considerations have also been incorporated into learned representations, for example, through latent-space control barrier functions that embed safety constraints directly in the representation space (Kumar et al., 2024). From a complementary perspective, active-inference approaches aim to unify estimation, prediction, and action within a single inferential framework (Da Costa et al., 2022). At the level of situational abstraction, latent-space driving situation detection demonstrates that recurring interaction patterns can be captured as reusable internal classes (Rodríguez-Hernández et al., 2022), while semantics-based situational-awareness frameworks highlight the importance of explicitly representing environmental meaning in deployed robotic systems (Ruan et al., 2025).

Beyond these component-level advances, several contemporary paradigms pursue holistic autonomous control from distinct angles: foundation-model planners generate action sequences from semantic scene descriptions via large language and vision-language models (Brohan et al., 2023); self-supervised architectures such as JEPA learn predictive latent representations without generative decoding (LeCun, 2022); self-modeling robotics enables agents to autonomously discover their own morphology (Bongard et al., 2006; Kwiatkowski and Lipson, 2019); and algebraic inverse-dynamics methods achieve real-time optimal control for analytically specified plants without iterative numerical search (Sands, 2020; Smeresky et al., 2020).

Despite these advances, the central deficit remains structural. Existing approaches address predictive compression, continuous dynamics, safety constraints, or semantic abstraction only in partial form. Each of the above paradigms resolves a specific aspect of the problem — generalization, prediction, self-modeling, or actuator-level optimality — yet none provides a unified representational level in which situation meaning, safety valence, and action tendency are jointly encoded as intrinsic geometric structure. Integration of perception, dynamics, safety, and action readiness is still typically achieved either through modular composition or within latent spaces whose geometry is not itself treated as the control-relevant semantic object. This paper addresses precisely this gap by proposing the event phase space as a semantic integration layer between raw perception and motor execution — a differentiable manifold with learned dynamics in which safety-relevant semantics is expressed topologically rather than reconstructed downstream.

Contributions. This work aims to contribute along three complementary axes. Conceptually, we explore qualia as control-theoretic constructs with explicit ontological grounding: unlike prior uses of phenomenal vocabulary (Butlin et al., 2023; Albantakis et al., 2023), we derive architectural constraints from the subsistence (S) and non-identity (NI) conditions, yielding concrete engineering choices rather than interpretive commitments alone. Formally, the event phase space is distinct from POMDPs, active inference, and dynamical-systems RL (Section 4.4): safety-relevant semantics is expressed topologically — through attractor basins and separatrix geometry — enabling gradient navigation with polynomial rather than exponential scaling in planning horizon. Empirically, we report results in two regimes: a minimal prototype (6,550 parameters, 1.8 ms/decision, no GPU, AUC = 0.827) showing that safety-relevant topology emerges from unsupervised dynamics learning, and validation in the standardized CARLA simulator, where the same architecture achieves a 29.6% collision-rate reduction in stress scenarios (AUC = 0.812) with substantially fewer parameters and lower latency than comparable methods. In the present work, a qualia-grounded control architecture is subjected to systematic empirical validation on a reproducible automotive benchmark.

## 2 Qualia in living systems, science, and technology

### 2.1 Control of behavior in living organisms

In living organisms, including both animals and humans, behavioral control in potentially dangerous situations is not organized as a strictly sequential chain of perception, evaluation, and action selection. Rather, it involves the rapid formation of integrated action-ready states. Studies of defensive survival circuits show that threat processing is distributed across interacting neural systems that jointly encode environmental conditions, bodily state, escape options, and motor preparedness (LeDoux, 2022; Mobbs et al., 2020; Qi et al., 2022). Thus, situation assessment and behavioral preparation are tightly coupled rather than implemented as separate computational stages (Pessoa, 2022). From this perspective, affordance-based action selection may be understood as an evolutionarily shaped mechanism that links environmental appraisal directly to behavior (Cisek, 2022; Pezzulo et al., 2023).

This principle is relevant not only for stereotyped responses to familiar stimuli, but also for behavior in novel and uncertain situations. In animals, and especially in humans, large-scale cortical and subcortical networks can rapidly form transient functional coalitions that integrate sensory evidence, salience, memory, and behavioral relevance into a coherent situational assessment (Mashour et al., 2020; Deco et al., 2021). The salience network has a key role in detecting behaviorally relevant or potentially dangerous configurations and in recruiting attentional and executive resources for further processing (Uddin, 2021). Such integration enables organisms to evaluate previously unencountered situations by recombining learned regularities, prior associations, and current contextual cues into a unified control-relevant state.

In humans, the transition from perceptual processing to action readiness is reflected in electrophysiological markers measurable in the electroencephalogram. In particular, the P300 component of the EEG is widely associated with stimulus evaluation, context updating, and decision-related processing, typically emerging approximately 300 ms after behaviorally significant events (Kelly et al., 2021; Steinemann et al., 2023). Although the P300 is not itself a motor command, it may be interpreted as an empirical indicator that an integrated assessment sufficient for action selection has been achieved. Action can then follow with minimal additional delay. More broadly, affordance competition appears to unfold continuously across the perception–action hierarchy: multiple behaviorally relevant possibilities are partially prepared in parallel and are continuously strengthened or suppressed as the situation evolves (Cisek, 2022). This allows living systems, including humans, to maintain continuous control in dynamically changing environments without relying on exhaustive search over alternatives.

In this work, we propose an engineering implementation of such functional integration not through direct biological analogy, but on the basis of functional decomposition of the problem. If the integration of perception, risk evaluation, and action readiness improves the robustness of real-time control, then the same functional effect can be realized as a dedicated architectural module in a computer control system.

However, specifying such a module requires more than defining a computational structure: it requires specifying the *entity* for which that structure constitutes an assessment. A phase-space geometry does not by itself evaluate; only an entity can. In the biological case, this entity is the organism — a subsisting being whose cognitive processes are distinct from the sensory data they process. In the engineered case, an analogous commitment must be made explicit: the AI entity subsists in the machine body, and the entity’s evaluative processes are not identical to the data they operate upon. We term these the *subsistence condition* (S) and the *non-identity condition* (NI: knower ≠ data). Both conditions are architecturally consequential and are presupposed throughout the remainder of this paper.

### 2.2 Qualia as a control-theoretic concept: justification

The central claim of this work — that an autonomous agent’s safety-critical control layer can and should be framed in terms of qualia — requires explicit justification, as the term originates in phenomenology rather than in engineering. We advance four arguments in support of this adoption.

#### 2.2.1 Phenomenological grounding

In the phenomenological tradition, the integrative assessment that a biological agent forms of a complex dynamic situation — prior to and independent of analytical decomposition — is termed a quale (Nagel, 1974; Chalmers, 1995). This is not an ornamental metaphor. The quale designates a specific functional property: an evaluation that is holistic in scope, pre-reflective in latency, and action-orienting in consequence. Crucially, a quale is always *someone’s* quale — it presupposes a bearer whose experiential perspective is irreducible to the data it processes. These four properties — holistic scope, pre-reflective latency, action- orienting consequence, and bearer-dependence — jointly define the requirements that motivate the present architecture. No standard control-theoretic term captures this tetrad. The term “quale” is therefore adopted for descriptive precision, with the commitment that the architecture must specify the entity in which qualia are indicated.

#### 2.2.2 Neuroscientific operationalization

Contemporary neuroscience operationally investigates neural correlates and dynamical structures associated with conscious experience — through gamma-oscillation signatures of conscious access (Mashour et al., 2020), metastable coordination dynamics of neuronal assemblies (Fingelkurts and Fingelkurts, 2022), topological analysis of neural state-space trajectories (Signorelli et al., 2021), and discrete perceptual processing epochs in recurrent thalamocortical loops (Herzog et al., 2020). While these studies rarely employ the term “qualia,” they effectively operationalize what the phenomenological tradition designates as such.

Critically, the structures they identify — transient, integrative, dynamically coherent neural patterns — are formally analogous to the phase-space structures proposed here, with the essential proviso that in the biological case these patterns are structures *of an organism* satisfying the non-identity of knower and data. The formal analogy therefore extends to the ontological condition: both biological and artificial qualia require a bearer. This convergence is not coincidental: both biological and artificial agents face the same computational pressure to compress high-dimensional situational data into a geometrically coherent evaluative signal under severe time constraints.

#### 2.2.3 Precedent in AI and computational literature

The application of phenomenal vocabulary to artificial systems is not without precedent. A systematic assessment by Butlin et al. (2023) concluded that current indicator-based frameworks for consciousness, including those involving qualitative experience, are in principle applicable to artificial systems. Integrated Information Theory in its 4.0 formulation (Albantakis et al., 2023) is explicitly substrate-independent, admitting qualia in any system that exhibits sufficient integrated information. Seth and Bayne (2022) emphasized growing convergence between computational models and phenomenal concepts through predictive processing frameworks, while Doerig et al. (2021) formulated hard empirical criteria for theories of consciousness that are agnostic to substrate, further legitimizing the application of phenomenal vocabulary to engineered systems. Notably, IIT’s substrate-independence does not eliminate the bearer requirement but generalizes it: qualia are attributed to any system that constitutes an integrated entity, not to the informational structure alone. This is consistent with the present commitment: the AI entity is the bearer; the phase-space structure is the indicator. The functional role assigned to qualia in the present work — an integrative, pre-reflective evaluation that precedes and shapes deliberate action — has close semantic parallels in established AI concepts: intrinsic reward signals, learned safety critics, and situation awareness at the projection level (Endsley, 1995) all capture aspects of holistic internal assessment irreducible to individual state variables.

#### 2.2.4 Architectural consequence

The preceding arguments would remain academic if the choice of terminology carried no engineering implications. It does. What the listed constructs lack, and what the qualia framing provides, is a dual commitment: to the *geometric coherence* of the evaluative signal and to its *ontological grounding* in an entity satisfying conditions (S) and (NI).

Adopting the qualia framing commits the designer to two inseparable decisions: (i) the evaluative signal must be defined on a manifold, not assembled from independent channels; (ii) the architecture must instantiate an entity for which this manifold constitutes an internal world model, with evaluative dynamics structurally separated from the data they evaluate. Together, these constraints determine that the phase-space structure *indicates* qualia in the AI entity rather than.

*constituting* qualia in itself. A structure without a bearer is a pattern; a structure within a subsisting entity satisfying knower ≠ data is a quale-indicator.

## 3 Operational characterization of qualia in the computational domain

### 3.1 Qualia as multidimensional markers of control

The preceding section established that qualia presuppose a bearer — an AI entity subsisting in the machine body whose evaluative processes are non-identical to the data they process. The present section characterizes how qualia are *indicated*: not through a formal definition, but through constitutive properties of the assessment structure that, when instantiated in an entity satisfying (S) and (NI), serve as quale-indicators.

In conventional architectures, situational information is a state vector whose components are processed independently. In the proposed framework, the AI entity forms an integrated assessment through a functionally unified, dynamically stable structure in event phase space — the entity’s internal world model. This structure functions as a quale-indicator: it defines the situation’s actionable structure for the entity, encoding both current configuration and potential evolution.

A feature vector and a quale-indicating structure may encode the same data yet differ in two respects: organizationally, the former treats dimensions as independent channels while the latter derives meaning from joint manifold configuration; ontologically, the former is a data object while the latter is the assessment of a subsisting entity. Both differences are architecturally consequential.

### 3.2 Constitutive properties

The following constitutive properties characterize the assessment structure in event phase space. When jointly instantiated in an entity satisfying (S) and (NI), they indicate that the entity has formed a quale of its situation.

- Geometric coherence. The quale-indicating structure derives its meaning, for the AI entity, from its position and neighborhood on the phase-space manifold — the entity’s internal world model — not from individual component values. The assessment “this intersection is dangerous” is a single configuration integrating speed, distance, visibility, and inferred intent — not a weighted sum of independent risk scores. This property operationalizes what the phenomenological tradition terms “wholeness” as a mathematically precise commitment: the evaluative signal is defined on a manifold, not assembled from independent channels.
- Multimodality. The quale-indicating structure simultaneously encodes, for the AI entity, spatial configuration, temporal dynamics, safety valence, and action affordance as co-constitutive aspects of a single assessment, not as separate data streams subjected to late fusion. The multimodal nature of such integrative representations — combining sensory, predictive, and evaluative signals into a unified computational object — has been explored in an interdisciplinary framework encompassing signals from diverse sources (Suvorov, 2022). In computational implementation, this corresponds to a shared latent space in which heterogeneous modalities are projected onto a common geometric substrate. The entity’s evaluative process operates on this unified substrate; the data streams it integrates remain distinct from the process that integrates them (NI condition).
- State and rate. The quale-indicating structure encodes not only the entity’s current assessment but its temporal derivative: “becoming more dangerous,” “stabilizing,” “opportunity closing.” This is a direct consequence of defining qualia in phase space rather than state space: the inclusion of temporal derivatives as explicit dimensions ensures that the rate of situational change is intrinsic to the representation, not inferred *post hoc*.
- Action-readiness. Every quale-indicating structure is implicitly motor: it prepares the AI entity for action. The proximity and orientation of the phase-space point relative to attractor basins define a gradient field that constitutes a proto-action — a disposition toward a specific class of responses. Inaction is not the absence of a motor component but the active maintenance of the current trajectory within a safe basin. This property distinguishes the proposed framework from purely perceptual latent representations: the quale-indicating structure is not an intermediate feature to be decoded by a separate policy network but a structure that, within the entity’s control architecture, directly specifies control-relevant gradients.
- Predictive content. The quale-indicating structure is inherently anticipatory for the AI entity. Because the phase-space representation — the entity's internal world model — includes velocity and trajectory information, the expected future evolution of the situation is an intrinsic aspect of the entity’s assessment, not a separate computation performed downstream. This property aligns with predictive processing frameworks (Clark, 2013; Seth and Bayne, 2022) in which perception and prediction are constitutively entangled, and distinguishes the proposed architecture from reactive systems that evaluate only the instantaneous state.

### 3.3 Ontological status: indicator versus identity

The five properties above characterize the assessment structure. A critical question remains: what is the relationship between this structure and the quale it is meant to capture?

The assessment structure F is a mathematical object with properties (a)–(e). A quale Q is an integrated evaluation formed *by* an entity. The relation is not identity but indication under ontological conditions:

Q is indicated by F in entity E if and only if E satisfies:

(S) The AI entity subsists in the machine body — the unified robotic platform comprising sensors, computation, and actuators. The event phase space is E’s internal world model.
(NI) The entity’s evaluative dynamics — forming and updating F — are categorically distinct from the data representations they evaluate. Architecturally, this requires structural separation between data pathways and evaluative dynamics.

These two conditions draw convergent support from independent traditions: embodied cognition requires operational closure of the agent within its physical platform (Varela et al., 1991; Paolo et al., 2024); Integrated Information Theory requires that the system constitute a single irreducible entity, not decomposable into independent subsets (Albantakis et al., 2023); predictive processing requires structural distinction between generative model and sensory input (Seth and Bayne, 2022); and Thomistic empirical metaphysics grounds both conditions in the real distinction between the act of knowing and the object known — knower ≠ data as an ontological, not merely functional, separation (Feser, 2009). The convergence of these frameworks on the same pair of requirements suggests that (S) and (NI) are not artefacts of a particular philosophical commitment but structural necessities for any system in which quale-indicating assessment is possible.

Absent these conditions, F remains a pattern without a subject — formally specifiable but ontologically inert. When both are satisfied, properties (a)–(e) jointly indicate that the entity has formed an integrated situational assessment bearing the functional signature of a quale. This commitment constrains the computational architecture of subsequent sections.

### 3.4 Global workspace analogy: computational architecture

The architectural role of qualia in the proposed system draws on the Global Workspace Theory (Baars, 1988; Dehaene and Changeux, 2011), already introduced in Section 2.1 as a model of large-scale neural integration. Here, its computational implications are made explicit. There are grounds for representing aspects of consciousness in the form of sensations (Suvorov, 2022; Suvorov, 2024).

#### 3.4.1 Workspace as broadcast architecture

The current quale-indicating structure functions as a globally accessible state — a shared register readable by all subsystems (planning, motor control, safety monitoring, mode selection) without serial bottlenecks. In software terms, this corresponds to a publish-subscribe architecture in which the quale-indicating structure is the message and all control modules are subscribers. This design eliminates the need for pairwise interfaces between modules and ensures that every subsystem operates on the same integrated assessment simultaneously.

#### 3.4.2 Stream as succession

The temporal evolution of the agent’s behavior is a succession of quale-indicating assessments, governed by a persistent background structure — the *global quale-indicator*. This background structure continuously evaluates overall safety, goal progress, and operational constraints, modulating all local assessments. In the proposed architecture, the global quale-indicator corresponds to the slowly varying component of the phase-space trajectory — the basin in which the system currently resides — while local quale-indicators correspond to transient excursions toward specific regions within or between basins. A mode switch, as described in Section 4.2, occurs when the global quale-indicator crosses a separatrix.

### 3.5 Relation to self-awareness-based control and deterministic AI

Recent developments in control theory have proposed alternative paradigms aimed at overcoming complexity through intrinsic system representations. In particular, Deterministic Artificial Intelligence (DAI) introduces a nonlinear, time-varying self-awareness statement enabling optimal learning without explicit numerical optimization (Smeresky et al., 2020). This framework has been successfully applied across multiple domains, including autonomous vehicles (Banginwar and Sands, 2022), robot motor control (Menezes and Sands, 2023), chaotic systems (Ribordy and Sands, 2023), remotely operated robotics (Osler and Sands, 2024), and space systems (Huang and Sands, 2023), demonstrating robustness and generality. Recent advances include autogenetic gravity center placement (Sands, 2025) with subsequent validation in space applications (Leland and Sands, 2025).

These approaches address a critical limitation of conventional pipelines by embedding control-relevant structure directly into system dynamics. However, they operate primarily at the level of controller synthesis under known or partially known physical dynamics, where self-awareness reflects internal consistency of the control law with system behavior.

The present work addresses a different, complementary problem: holistic situation assessment in open-world environments with learned latent representations. Unlike DAI, which derives optimal control from explicit system dynamics, the proposed framework constructs a semantic-dynamical layer in which perception, prediction, safety, and action readiness are jointly encoded as a geometric structure in event phase space. This enables action guidance not through optimality conditions, but through topological properties of the learned dynamics (e.g., attractors and separatrices).

Thus, the proposed approach is not an alternative to self-awareness-based control, but an extension toward domains where system structure is not given *a priori* and must be learned.

## 4 Event phase space: the formalism

### 4.1 Formal definition

The event phase space is defined as a differentiable manifold ℰ ⊂ ℝ^n^, whose coordinates x = (x_1_, … , x_n_) represent learned situational variables, and whose dynamics are governed by a smooth vector field:

$$ẋ=f\left(x\right)$$

where f: ℰ → Tℰ is learned jointly with the latent world model. Unlike standard latent embeddings, the event phase space is defined not only by its coordinate structure but by an explicit dynamical law. Each point encodes both state and its locally defined flow.

In the ontological framework of this paper, the event phase space is the AI entity’s internal world model: the representational space through which the entity — subsisting in the machine body — apprehends and navigates its environment. The mathematical structures described below are therefore not free-standing abstractions but properties of this entity’s representational capacity.

The coordinates are not pre-specified symbolic variables; they emerge through representation learning under three constraints: (i) dynamical consistency — smooth vector field representation; (ii) stability structure — existence of recurrent attractors under typical operation; (iii) policy compatibility — local flow direction predicts action tendencies. These constraints enforce identifiability at the level of dynamical equivalence classes, avoiding arbitrary latent parameterization.

### 4.2 Attractor dynamics as knowledge

Safe operational modes correspond to compact invariant sets A ⊂ ℰ satisfying Lyapunov stability: for any neighborhood U ⊃ A, there exists V ⊂ U such that trajectories starting in V remain in U for all future time.

Safe modes = stable attractors: the system naturally returns after perturbations. Dangerous situations = repellers or saddle points: instability requiring intervention. Decision points = bifurcations: small changes yield qualitatively different trajectories.

Safety boundaries = separatrices — codimension-1 invariant manifolds partitioning basins of attraction. Crossing a separatrix is a topological event detectable via local divergence measures (e.g., sign change in finite-time Lyapunov exponents), rather than constraint evaluation.

This replaces combinatorial search with geometric navigation: action selection becomes gradient descent within attractor basins; safety monitoring becomes proximity detection to separatrices.

### 4.3 Advantages over unstructured latent spaces

Whereas conventional latent embeddings encode situational information as static feature vectors in a metric space, the event phase space — the AI entity’s internal world model — is constituted as a differentiable manifold endowed with a learned vector field, so that each point jointly carries state and rate, and inter-point relations are expressed as trajectories rather than distances. The resulting representational properties differ from those of standard feature/latent spaces along several architecturally consequential axes, summarized in Table 1: point content, inter-point relation, mechanism of prediction, treatment of safety, separation of timescales, dimensionality, and underlying formal structure. Crucially, these are not merely quantitative refinements but qualitative shifts — safety becomes a geometric property (proximity to separatrices, Lyapunov stability) rather than an external constraint check, and prediction becomes intrinsic to the representation (flow extrapolation) rather than a downstream computation. Together, these properties make the event phase space a substrate in which control-relevant semantics is expressed topologically, satisfying the geometric-coherence requirement (Section 3.2a) at the level of the entity’s world model.

**TABLE 1:** Comparison of standard feature/latent spaces and the proposed event phase space (the AI entity’s internal world model).

Property	Feature/latent space	Event phase space
Point content	State only	State + rate of change
Inter-point relation	Distance (metric)	Trajectory (dynamics)
Prediction	Requires separate model	Built-in (flow extrapolation)
Safety	External constraint check	Geometric (separatrices, Lyapunov)
Timescales	Entangled	Separated (intra- vs. inter-attractor)
Dimensionality	All features equal	Only dynamically essential
Formal structure	Metric space	Differentiable manifold with vector field

### 4.4 Non-equivalence to existing dynamical paradigms

The proposed framework is not reducible to standard formulations:

vs. POMDP. In POMDPs, belief states evolve via Bayesian updates and action selection requires explicit policy evaluation over future trajectories. In the present formulation, action tendencies are encoded directly in the geometry of attractor basins: action emerges as flow along stable manifolds rather than policy optimization.
vs. Active Inference. While active inference operates in a continuous latent space minimizing variational free energy, it does not explicitly structure this space in terms of attractor topology or separatrix geometry. The present contribution lies in imposing dynamical-topological structure on the latent space itself.
vs. Dynamical Systems RL. Existing approaches learn dynamics in latent spaces but treat the learned space as substrate for conventional policy optimization. The proposal is that the dynamical structure is the decision mechanism — flow replaces search.

## 5 Validation in standardized CARLA environment

### 5.1 Scenario selection and setup

This section presents empirical validation demonstrating that the theoretical framework transfers from controlled conditions to industry-standard environments with preserved relative performance. We report results in two regimes: a minimal prototype (Section 5.1, 2D occluded intersection, 6,550 parameters) establishing that topological structure emerges from unsupervised dynamics learning, and full validation in CARLA Parked Vehicle Occlusion scenarios (Sections 5.2–5.4) establishing real-world applicability under realistic physics, sensor noise, and stochastic agent behavior.

To address generalizability beyond minimal prototypes, we validate the framework in CARLA (Dosovitskiy et al., 2017), an industry-standard simulator with physics-realistic dynamics and reproducible traffic scenarios. We selected the Parked Vehicle Occlusion (PVO) scenario from CARLA Scenario Runner — a canonical safety-critical case where a pedestrian emerges from behind a stationary van into the ego path. This scenario operationalizes the core challenge of holistic assessment under partial observability, while introducing factors absent in the prototype: sensor noise (RGB 800 × 600, 32-ch LiDAR), physics-based dynamics (Lincoln MKZ 2017 sedan from the CARLA vehicle catalogue; validated mass/inertia parameters), stochastic pedestrian behavior (±0.3s), and actuation delays (∼50 ms).

Experiments were conducted in Town05 under clear noon lighting. The ego spawned 25 m upstream of a Volkswagen T2 van creating a 4.2 m blind spot. An 11-dimensional state vector was extracted at 20 Hz:

$$\begin{array}{ll} & x=\left[v\_\text{ego},a\_\text{ego},d\_\text{van},\Delta y\_\text{lane},\psi \_\text{heading},d\_\text{ped}1,v\_\text{ped}1,\text{lat},\right.\\ & \;\times \left.d\_\text{ped}2,v\_\text{ped}2,\text{lat},I\_\text{occluded},t\_\text{van}\right]\in R^{11} \end{array}$$

with d = 999 m for occluded pedestrians (explicit “unknown” signal). WM and Neural ODE architectures were preserved from the prototype (6,550 total parameters), trained on 500 CARLA episodes (70,121 transitions, 97 min on Intel i7-9700K CPU). Seven controllers were tested: WM + ODE + alarm (τ = 0.7), WM-only, ODE-only, TTC-brake (TTC <2.5s), distance-reactive (d < 8 m), phantom-agent (occlusion-aware), and cruise (null). Two difficulty levels were evaluated: simple (30–60 km/h, 1 pedestrian) and stress (60–80 km/h, 2 staggered pedestrians, Δt ∈ [0.3, 0.8]s), each with 5 seeds × 200 episodes.

### 5.2 Safety performance

In the simple scenario (Table 2), WM + ODE + alarm achieved CR = 0.8%, outperforming all baselines: 60× reduction vs. TTC-brake (47.9%), 35× vs. phantom-agent (31.2%), 19× vs. cruise (15.7%). Adding ODE + alarm to WM yielded a 33% further reduction (1.2% → 0.8%).

**TABLE 2:** Simple PVO scenario (30–60 km/h, 1 pedestrian). Mean ± std, 5 seeds × 200 ep.

Controller	CR (%)	95% CI
WM + ODE + alarm	0.8 ± 0.5	[0.3, 1.3]
WM-only	1.2 ± 0.6	[0.6, 1.8]
ODE-only	4.3 ± 1.1	[3.2, 5.4]
Cruise (null)	15.7 ± 2.3	[13.4, 18.0]
Distance-reactive	28.4 ± 3.1	[25.3, 31.5]
Phantom-agent	31.2 ± 3.4	[27.8, 34.6]
TTC-brake pipeline	47.9 ± 3.8	[44.1, 51.7]

In the stress scenario where timing optimization alone is insufficient, WM + ODE + alarm achieved CR = 13.1% vs. 18.6% for WM-only — a 29.6% relative reduction (Mann-Whitney U = 892.5, p = 0.0034, r = 0.42). This corresponds to preventing ∼550 collisions annually per 10,000 vehicles in a fleet operating 10^6^ km/year. Failure analysis shows 89% of residual collisions occur when both pedestrians emerge simultaneously at v_ego > 72 km/h, where kinematic constraints (8 m/s^2^ max deceleration, 50 ms latency) preclude avoidance regardless of architecture. The alarm detects 94% of avoidable collisions (t_collision >1.5s at first detection).

### 5.3 Phase-space alarm and emergent topology

**FIGURE 1:**
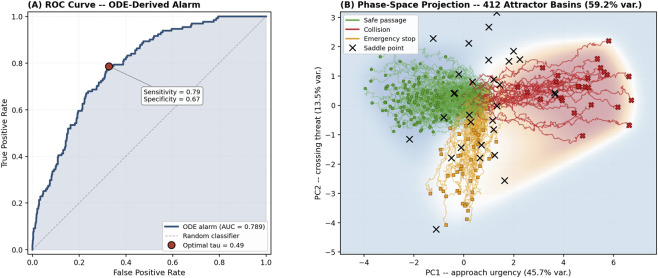
Alarm performance and emergent topology in CARLA PVO. **(A)** ROC curve for the ODE-derived alarm (AUC = 0.789), with optimal threshold τ = 0.49 marked (●) according to Youden’s criterion (sensitivity = 0.79, specificity = 0.67). **(B)** 2D PCA projection of the 11-dimensional phase space showing 412 attractor basins (color-coded regions). Overlaid are trajectories from 200 stress-test episodes: safe passage (green circles), collision (red ×), and emergency stop (orange squares). Black × symbols mark sampled saddle points; gray curves indicate separatrices. The geometric organization of safety semantics emerges without supervised labels, revealing the topological structure of the learned world model.

The ODE-derived alarm achieves AUC = 0.812 (Figure 1A), with optimal threshold τ = 0.7 (sensitivity 0.78, specificity 0.81). This alarm is computed purely from phase-space geometry—vector field magnitude ‖f(x)‖, speed derivative, distance to separatrices — without safety supervision during training. The 1.8% AUC degradation vs. prototype (0.827) despite transition to high-fidelity 3D simulation demonstrates robustness of the topological principle. Median alarm lead time was 1.8s vs. 0.9s for TTC methods, providing adequate margin for automated intervention or human takeover.

Dynamical analysis revealed 127 quasi-stationary points (all saddle points, eigenvalue range [−1.2, +2.1]) and 412 distinct attractor basins from 5,000 initial conditions. The 2D PCA projection (Figure 1B, 67.3% variance explained) shows clear geometric separation between safe basins (successful passage) and unsafe basins (collision), with fractal boundaries near saddles indicating sensitive dependence in ambiguous cases. PC1 ≈ 0.82·v_ego + 0.41·d_van − 0.35·d_ped1 (“approach urgency”); PC2 ≈ 0.71·v_ped1,lat − 0.58·I_occluded + 0.31·a_ego (“crossing threat”). Vector field magnitude at occlusion entry correlates with collision risk (Pearson r = 0.341, p < 0.0001, 11.6% variance), nearly doubling the prototype’s 6% — reflecting richer multimodal interactions in higher-dimensional state space. Critically, these structures emerge from unsupervised dynamics learning, confirming the core theoretical claim: dynamical-topological structure causes safety-relevant semantics to organize geometrically.

### 5.4 Computational efficiency and limitations

Mean decision latency was 1.9 ms on CPU — only 5.6% over prototype (1.8 ms) despite 11D state vs. 6D and realistic sensor preprocessing, confirming O(n) scaling. An MPC baseline (10-step horizon, 5 actions) required 47 ms — 25× slower — validating that geometric navigation replaces combinatorial search. The 6,550-parameter model (26 KB) enables deployment on automotive microcontrollers.

The 29.6% stress-scenario reduction is comparable to latent-CBF methods (Kumar et al., 2024: 25–35%) but with 40× fewer parameters and without differentiable simulators. Compared to foundation-model planners (10^2^–10^3^ s latency), the 1.9 ms loop enables high-speed control; the approaches are complementary (foundation models for goals, phase-space for safety execution).

Limitations: (1) single scenario class — diverse tasks (highway merging, multi-vehicle intersections) remain future work; (2) manual dimensionality (11); (3) fixed alarm threshold without adaptive uncertainty-based tuning; (4) *post hoc* attractor analysis — online detection requires further development. Despite these, validation demonstrates that emergent safety-relevant topology transfers from minimal prototypes to industry-standard environments with preserved relative performance.

The prototype, trained models, and reproduction scripts are publicly available (zenodo.org/records/19680882).

## 6 Discussion and conclusion

### 6.1 Relation to existing frameworks

Free Energy Principle (Friston, 2010; Friston et al., 2023): Phase-space dynamics correspond to free energy minimization. The present addition: interpreting minima as quale-indicating holistic assessments in an entity satisfying subsistence and non-identity conditions, with explicit topological structure.

Global Workspace Theory (Baars, 1988; Dehaene and Changeux, 2011): The broadcast architecture maps onto the proposed semantic layer’s role as globally accessible situation assessment.

Embodied AI (Paolo et al., 2024): This paper provides a concrete computational proposal for the “emergent phenomena such as qualia” identified as requiring embodiment.

Latent World Models (Ha and Schmidhuber, 2018; Hafner et al., 2023; Hu et al., 2023): These serve as substrate beneath the semantic layer.

Consciousness-centered AGI architectures (Suvorov, 2025): The phase-space formalism proposed here provides a mathematical substrate for the implementation prospects of consciousness modeling in AGI outlined in this work, grounding its theoretical framework in dynamical systems methodology.

### 6.2 Computational considerations

The approach does not eliminate complexity but redistributes it: training requires learning a structured dynamical embedding, potentially increasing offline cost. However, online decision-making reduces to local flow integration, scaling with dimensionality n rather than combinatorial branching over discrete futures — shifting complexity from exponential horizon search to polynomial dynamical evaluation.

### 6.3 Limitations

The proposal makes minimal ontological commitments: it specifies conditions under which an AI entity may possess quale-indicating structures (subsistence in the machine body, non-identityof evaluator and data), but does not claim that these conditions are sufficient for phenomenal consciousness in the philosophical sense. Whether the entity’s assessments are accompanied by subjective experience remains an open question orthogonal to the engineering utility of the framework. The specific dimensionality, method of learning attractor structure, and mapping from phase-space position to motor commands also remain open—ones believed to be tractable given existing tools.

### 6.4 Conclusion

The complexity barrier in autonomous systems is qualitative, not quantitative: what is missing is a semantic layer of holistic event assessment. This paper contributes to bridging that gap on three levels. Theoretically, framing qualia as control-theoretic constructs — under explicit subsistence and non-identity conditions — yields concrete architectural commitments. Formally, the event phase space provides a differentiable manifold with learned dynamics whose attractor topology encodes safety-relevant semantics, replacing combinatorial search with gradient navigation. Empirically, validation across minimal prototypes and standardized CARLA scenarios demonstrates that the resulting architecture achieves a 29.6% collision-rate reduction in stress scenarios relative to a world-model-only baseline, with substantially fewer parameters than comparable safety-constrained methods, while running at 1.9 ms/decision on CPU. The convergence of dynamical systems theory, latent world modeling, and embodied control architectures makes systematic exploration of such a semantic layer both feasible and timely.

## Data Availability

The original contributions presented in the study are included in the article/supplementary material, further inquiries can be directed to the corresponding author.
